# Supplementary material for: The ontogeny of myeloid-stromal synovial tissue niches in rheumatoid arthritis
Source: Res Sq. 2025 Nov 21:rs.3.rs-8079358. Preprint. [Version 1] doi: 10.21203/rs.3.rs-8079358/v1 (PMC12668173; doi:10.21203/rs.3.rs-8079358/v1)
Supplement: 1 — Supplementary Figure 1 Legend. Detailed atlas of macrophage cell clusters in human synovial tissue across different conditions. (A) Dataset as in Figure 1 is visualized as MiloR neighbourhood graphs where nodes represent neighbourhoods, coloured by the most represented cell type cluster (left) or by whether >45% of cells within a neighbourhood belong to one specific cluster or not (right). (B-D) The changes in synovial tissue myeloid cell composition between healthy controls (n = 11) and naïve-to-treatment RA (n=12) or resistant to c/bDMARDs treatments (n=11) and between active RA (naïve and resistant to treatment) and in RA in sustained remission (n=11) as in Fig.1. Data are visualized as MiloR neighbourhood graphs on the left, where nodes represent neighbourhoods, coloured by their log fold change across conditions. Neighbourhoods with non-differential abundance (FDR > 10%) are coloured white, and node size reflects the number of cells in each neighbourhood. On the right, a beeswarm plot displays the distribution of log fold change across conditions for neighbourhoods containing cells from different cell type clusters. Differential abundance neighbourhoods at FDR ≤ 10% are highlighted in colour. (E) Density plots showing MerTK expression in synovial tissue myeloid cell atlas. Supplementary Figure 2 Legend. Proportion of distinct synovial tissue myeloid cell clusters between joint conditions. (A) UMAP visualization of peripheral blood (PB; n = 5 healthy donors and n = 3 RA patients) and synovial tissue from RA patient samples with active disease, including n = 23 (12 naïve to treatment and 11 resistant to c/bDMARDs) and n = 11 RA in sustained remission (longer than 9 months), as well as from healthy controls (n = 11). Data represent integrated single-cell transcriptomic analysis of synovial tissue (ST) myeloid cells as in Fig.1. (B) Stacked plot illustrating the frequency of STM clusters in healthy and RA synovial tissue. (C) The proportion of different myeloid cel [file NIHPPRS8079358V1-supplement-1.pdf]

Supplementary Tables

Table S1: Demographic, clinical and immunological characteristics of Rheumatoid arthritis patients whose synovial tissue biopsies were used to generate synovial organoids

	**Active RA**<br>n=10
**Female sex, n(%)**	8 (80)
**Age, years (mean ± SEM)**	65.3 ± 3.1
**Disease Activity Score, (mean ± SEM)**	5.7 ± 0.4
**ACPA positivity, n(%)**	10 (100%)
**RF positivity, n(%)**	10 (100%)
**Concomitant treatments:**	Naive, n=4<br>c/b-DMARDs, n=6

ACPA: Anti-Citrullinated Peptide Antibodies; **RF:** Rheumatoid Factor.
c/b-DMARDs: conventional/biologic-Disease Modifying Anti-Rheumatic Drugs.

Table S2: Demographic, clinical and immunological characteristics of Rheumatoid arthritis patients whose synovial biopsies were used to extend synovial tissue myeloid cell atlas

	**Active RA**<br>n=4	**Sustained Remission RA**<br>n=5	**Healthy**<br>n=7
**Female sex, n(%)**	3 (75%)	3 (60%)	0 (0%)
**Age, years (mean ± SEM)**	55 ± 8.4	44 ± 2.1	37 ± 5.8
**Disease Activity Score, (mean ± SEM)**	5.3 ± 0.4	1.6 ± 0.2	N/A
**ACPA positivity, n(%)**	3 (75%)	4 (80%)	N/A
**RF positivity, n(%)**	2 (50%)	4 (80%)	N/A
**Concomitant treatments:**	Naive, n=2<br>c/b-DMARDs, n=2	c/b-DMARDs, n=5	N/A

ACPA: Anti-Citrullinated Peptide Antibodies; **RF:** Rheumatoid Factor.
c/b-DMARDs: conventional/biologic-Disease Modifying Anti-Rheumatic Drugs.

Table S3. Details of the antibodies used for sorting and phenotyping peripheral blood precursors and synovial organoid cells.

	Antibodies for sorting peripheral blood monocytes prior to organoid generation	Cat No./ Source
**1**	APC anti-human **HLA-DR**	307610 /Biolegend
**2**	Brilliant Violet 650 anti-human **CD14**	301830/Biolegend
**3**	Brilliant Violet 510 anti-human **CD16**	360730/Biolegend
**4**	PE/CY7 anti-human **CD88**	344308/Biolegend
**5**	AF700 anti-human **CD89**	354118/Biolegend
**6a**	FITC anti-human **CD15** (SSEA-1) dump channel	323004/Biolegend
**6b**	FITC anti-human **CD19**-dump channel	302206/Biolegend
**6c**	FITC anti-human **CD20**-dump channel	302304/Biolegend
**6d**	FITC anti-human **CD117** (c-kit)-dump channel	313232/Biolegend
**6e**	FITC anti-human **CD56** (NCAM)-dump channel	304604/Biolegend
**6f**	FITC anti-human **CD3**-dump channel	300440/Biolegend
	**Antibodies used for sorting/phenotyping fibroblasts and myeloid cells from synovial organoids**	**Cat No./ Source**
**1**	Brilliant Violet 711 anti-human **CD45**	304050/Biolegend
**2**	Brilliant Violet 785 anti-human **HLA-DR**	307642/Biolegend
**3**	Brilliant Violet 510 anti-human **CD64**	305028/Biolegend
**4**	AF700 anti-human **CD11b**	301356/Biolegend
**5**	APC anti-human **Folate Receptor b**	391706/Biolegend
**6**	PE anti-human **TREM2**	FAB17291P/R&D Systems
**7**	APC anti-human **SPP1**	50-9096-42/Invitrogen
**8**	PE/CY7 anti-human **MERTK**	367604/Biolegend
**9**	PE/Dazzle594 anti-human **CD48**	562717/BD Biosciences
**10**	PE/CY7 anti-human **CD90**	328124/Biolegend
**11**	PE/Dazzle594 anti-human **PDPN**	337028/Biolegend
**12**	AF700 anti-human **CD31**	303104/Biolegend

Table S4. Details of antibodies used for sorting DC subsets and monocytes from peripheral blood of healthy donors for SNP-based synovial organoid system

	Antibody	Cat No./ Source
**1**	Brilliant Violet 421 anti-human **CD5**/Brilliant Violet 421 anti-human **BTLA**	300626/Biolegend<br>344512/Biolegend
**2**	Brilliant Violet 510 anti-human **CD45RA**	304142/Biolegend
**3**	Brilliant Violet 605 anti-human **FcER1A**	334628/Biolegend
**4**	Brilliant Violet 650 anti-human **CD14**	301836/Biolegend
**5**	Brilliant Violet 711 anti-human **CD163**	333630/Biolegend
**6**	Brilliant Violet 785 anti-human **HLA-DR**	307642/Biolegend
**7**	PE anti human **CD141**	559781/BD
**8**	PE/CY5 anti-human **CD16**	302010/Biolegend
**9**	PE/CY7 anti-human **CD88**	344308/Biolegend
**10**	PE/Dazzle 594 anti-human **CD123**	306034/Biolegend
**11**	APC anti-human **CD1c**	331524/Biolegend
**12**	AF700 anti-human **CD89**	354118/Biolegend
**13**	PerCP_Cy5.5_anti-human **CD36**	336224/Biolegend
**14a**	FITC anti-human **CD15** (SSEA-1) dump channel	323004/Biolegend
**14b**	FITC antihuman **CD19** dump channel	302206/Biolegend
**14c**	FITC anti-human **CD117** (c-kit) dump channel	313232/Biolegend
**14d**	FITC antihuman **CD3** dump channel	300440/Biolegend
**14e**	FITC anti-human **CD56** (NCAM) dump channel	304604/Biolegend

Table S5. Primary and secondary antibodies used to map the fibroblast and immune cells in human synovial tissue biopsies, embryonic joints and synovial organoids

Primary Antibodies	Working Dilution / Concentration	Cat No./ Source	Secondary antibodies
Rabbit anti-human **COL1A**	1:100	HPA011795/Sigma	MP-7401/ ImmPRSS HRP Horse Anti Rabbit
Rabbit anti-human **COL3A**	1:100	HPA007583/Sigma	MP-7401/ ImmPRSS HRP Horse Anti Rabbit
Rabbit anti-human **PDPN**	1:200	HPA007534/Sigma	A-21245/ Goat anti-Rabbit IgG Alexa Fluor 647 (1:100)
Rabbit anti-human **MMP3**	1:50	ab52915 /Abcam plc	A-21245/ Goat anti-Rabbit IgG Alexa Fluor 647 (1:100)
Rabbit anti-human **CD31**	1:100	Ab76533 /Abcam plc	A-21245/ Goat anti-Rabbit IgG Alexa Fluor 647 (1:100)
Mouse anti-human **CD31**	1:200	Abab9498 /Abcam plc	A-212236/ Goat anti-Mouse IgG Alexa Fluor 647(1:100)
Rabbit anti-human **LYVE1**	1:200	HPA042953 / Sigma	A11008/ Goat anti-Rabbit IgG Alexa Fluor 448 (1:100)
Rabbit anti-human **SPP1**	1:100	ab101492/Abcam plc	A-21245/ Goat anti-Rabbit IgG Alexa Fluor 647 (1:100)
Rabbit anti-human **CD90**	1:100	Ab226123/Abcam plc	A-21245/ Goat anti-Rabbit IgG Alexa Fluor 647 (1:100)
Goat anti-human **TREM2** (Used in IF / Cell DIVE)	1:50	ab85851 /Abcam plc	A-21447/ Donkey anti-Goat IgG Alexa Fluor 647 (1:100)
Mouse anti-human **PRG4** (Used in IF / Cell DIVE)	1:100	MABT401/ Sigma	A-212236/ Goat anti-Mouse IgG Alexa Fluor 647(1:100) **(IF)**<br>A-21202/ Donkey anti-mouse IgG Alexa Fluor 448 (1:100) **(Cell DIVE)**
Mouse anti-human **CD45**	1:200	NBP2-44863/ Novus-Bio	A-21202/ Donkey anti-mouse IgG Alexa Fluor 448 (1:100)
Mouse anti-human **CD68**	1:40	M087629-2/ Dako, Cambridgeshire, UK	A-21202/ Donkey anti-mouse IgG Alexa Fluor 448 (1:100)
Rabbit anti-human **CD68** (Used in Cell DIVE)	10 µg/mL	ab280860 / Abcam plc	Directly conjugated to AF555
Mouse anti-human **CD31** (Used in Cell DIVE)	10 µg/mL	NBP2-33154AF647 / Novus-Bio	Directly conjugated to AF647

Table S6. Minimum and maximum exposure values used in QuPath analysis software for images displayed in Figure 2A-B

Antibody Target	Developmental Joint Minimum QuPath Exposure	Developmental Joint Maximum QuPath Exposure
PRG4	4000	1000
TREM2	6000	40000
CD68	5000	25000
CD31	5000	30000

Supplementary Figure 1 related to Figure 1

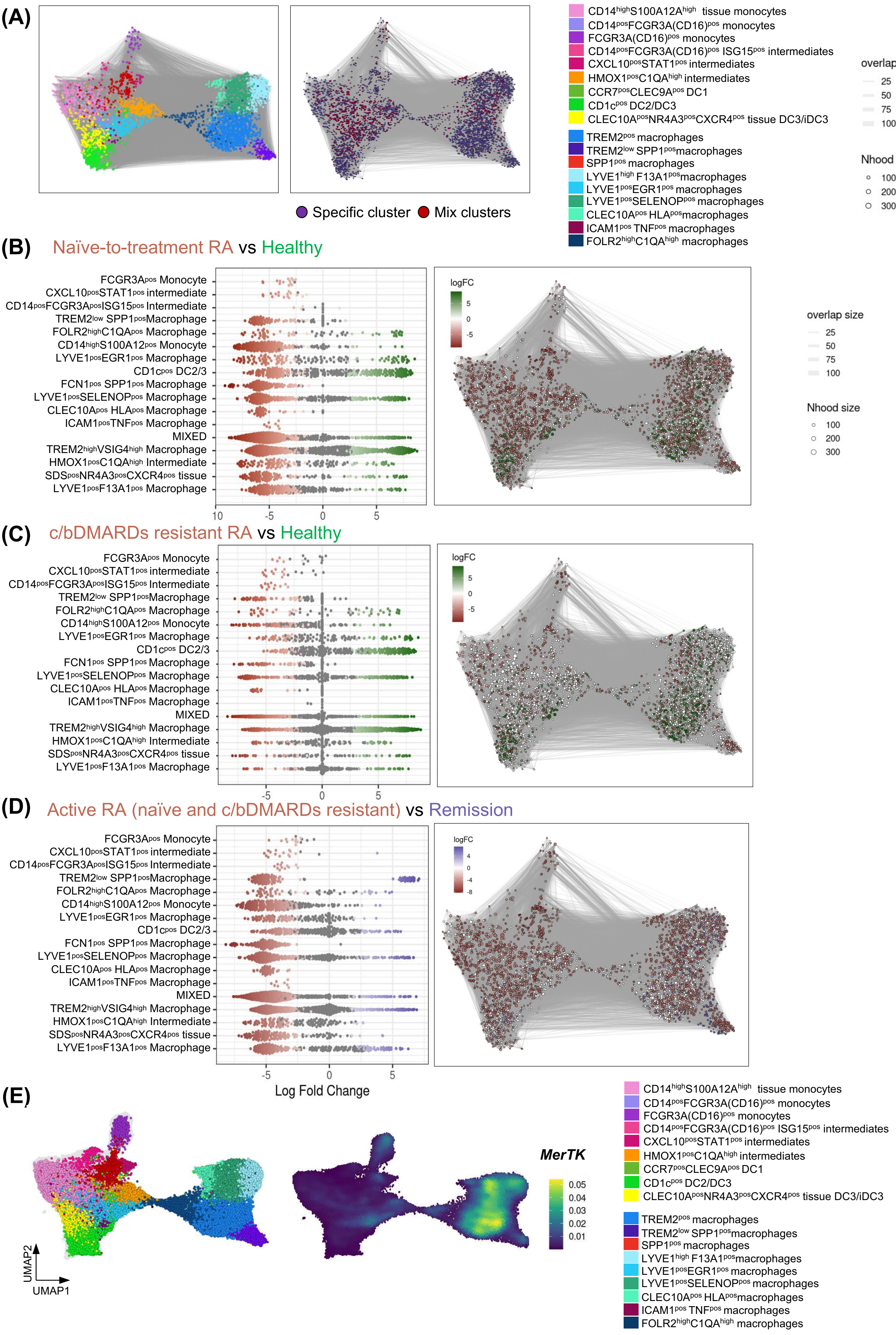

Supplementary Figure 1 Legend. Detailed atlas of macrophage cell clusters in human synovial tissue across different conditions.

(A) Dataset as in Figure 1 is visualized as MiloR neighbourhood graphs where nodes represent neighbourhoods, coloured by the most represented cell type cluster (left) or by whether >45% of cells within a neighbourhood belong to one specific cluster or not (right).

(B-D) The changes in synovial tissue myeloid cell composition between healthy controls (n = 11) and naïve-to-treatment RA (n=12) or resistant to c/bDMARDs treatments (n=11) and between active RA (naïve and resistant to treatment) and in RA in sustained remission (n=11) as in Fig.1. Data are visualized as MiloR neighbourhood graphs on the left, where nodes represent neighbourhoods, coloured by their log fold change across conditions. Neighbourhoods with non-differential abundance (FDR > 10%) are coloured white, and node size reflects the number of cells in each neighbourhood. On the right, a beeswarm plot displays the distribution of log fold change across conditions for neighbourhoods containing cells from different cell type clusters. Differential abundance neighbourhoods at FDR ≤ 10% are highlighted in colour.

(E) Density plots showing MerTK expression in synovial tissue myeloid cell atlas.

Supplementary Figure 2 related to Figure 1

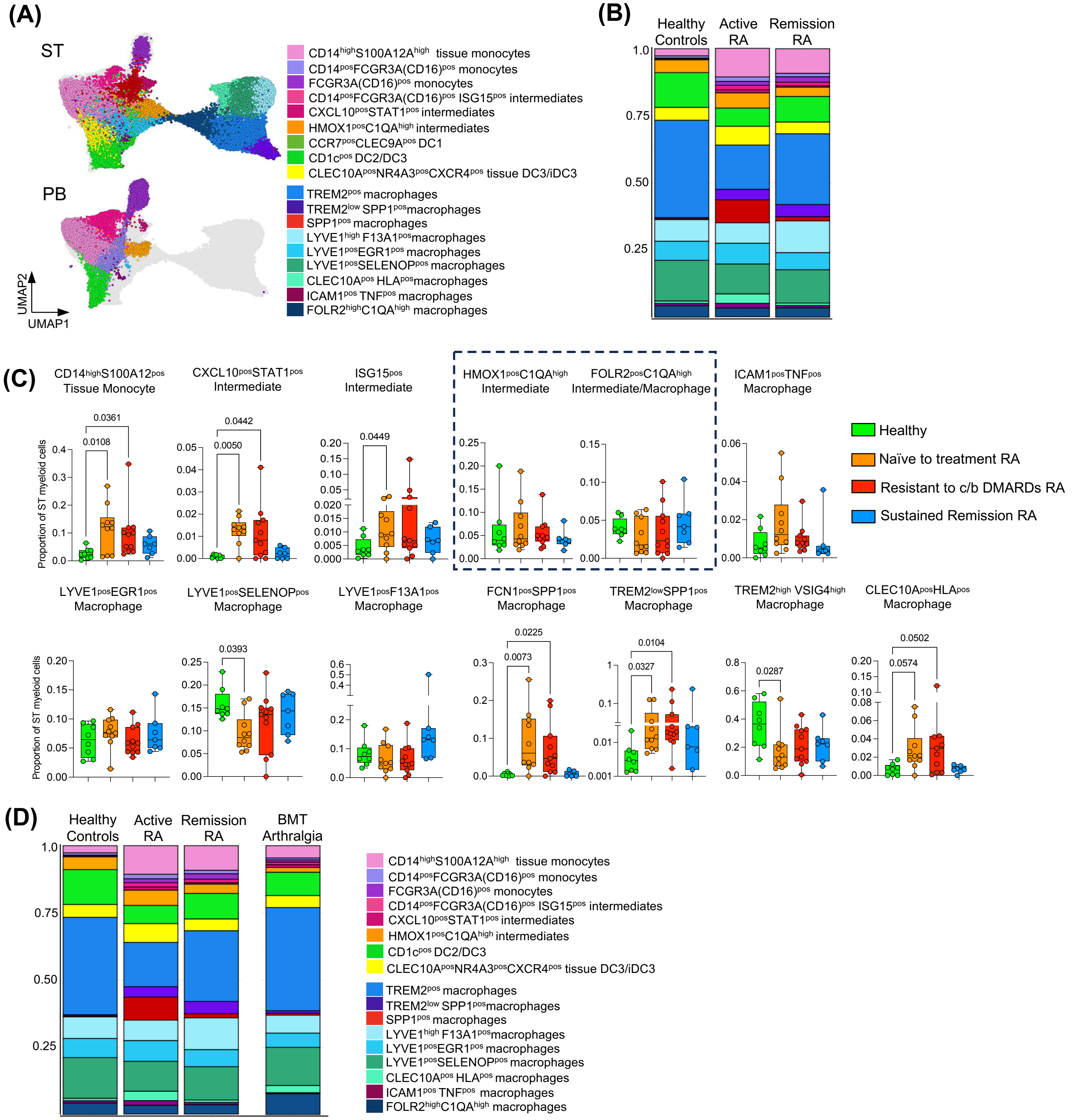

Supplementary Figure 2 Legend. Proportion of distinct synovial tissue myeloid cell clusters between joint conditions.

(A) UMAP visualization of peripheral blood (PB; n = 5 healthy donors and n = 3 RA patients) and synovial tissue from RA patient samples with active disease, including n = 23 (12 naïve to treatment and 11 resistant to c/bDMARDs) and n = 11 RA in sustained remission (longer than 9 months), as well as from healthy controls (n = 11). Data represent integrated single-cell transcriptomic analysis of synovial tissue (ST) myeloid cells as in Fig.1.

(B) Stacked plot illustrating the frequency of STM clusters in healthy and RA synovial tissue.

(C) The proportion of different myeloid cell clusters in synovial tissue differs between healthy controls, active RA, and RA in remission. Data are presented as boxplots showing the median and interquartile range; each dot represents an individual donor/patient. Statistical analysis was performed using the Kruskal–Wallis test with Dunn's correction for multiple comparisons. Exact p-values are shown on the graphs. The dotted box highlights two related clusters representing an intermediate state between tissue monocytes and macrophages, which in organoids are pooled into a single $C1QA^{pos}$ cluster.

(D) Stacked plot illustrating the frequency of STM clusters in a bone marrow transplant (BMT) patient in the context of the healthy and RA synovial tissue atlas.

Supplementary Figure 3 related to Figure 2 and 4

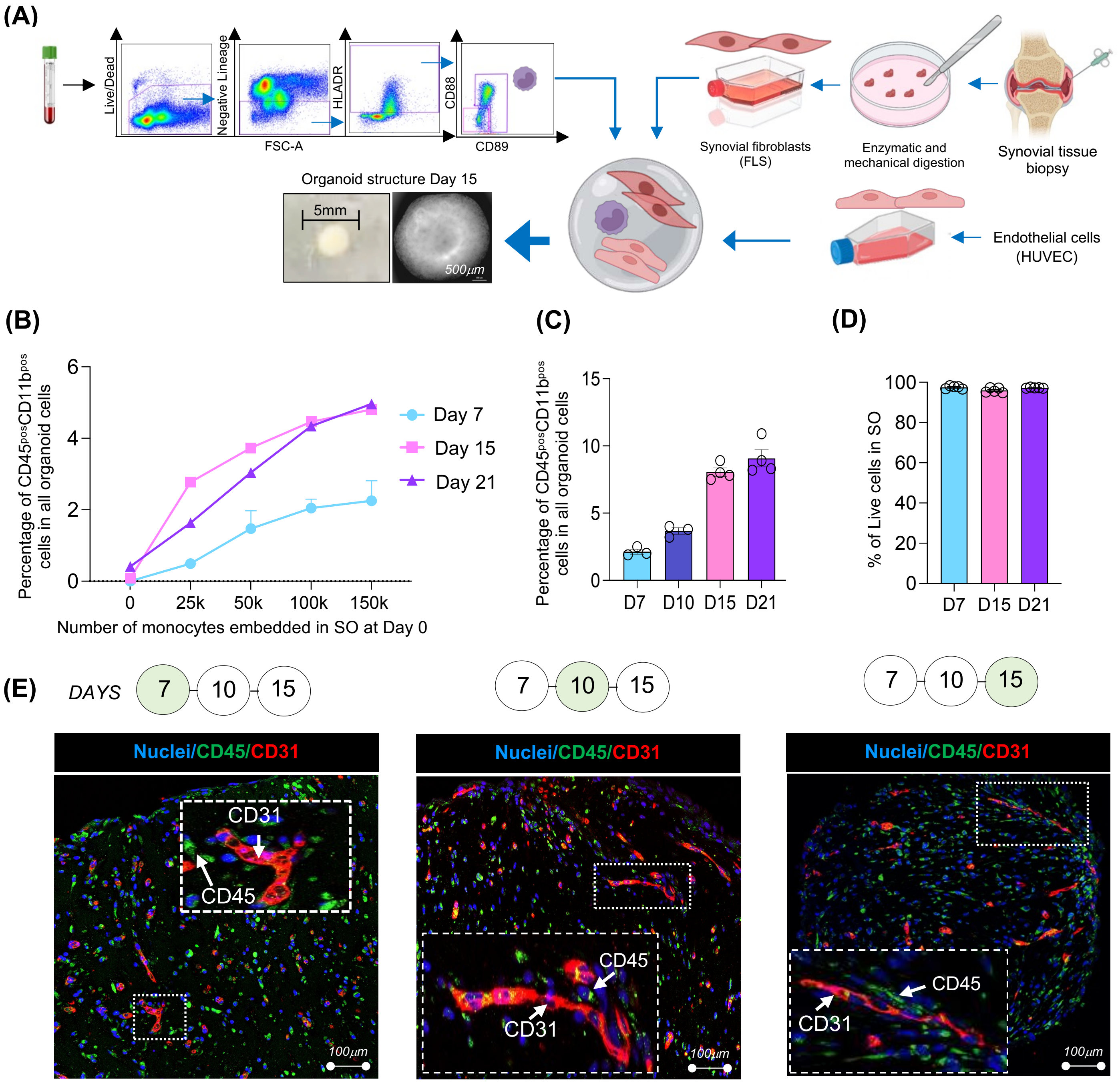

Supplementary Figure 3 Legend. Establishing a human myeloid-stromal synovial organoid system.

(A) Schematic illustrating the workflow of synovial organoid (SO) generation using synovial fibroblasts, blood monocytes, and endothelial cells in a single droplet of Matrigel as in Fig.2C.

(B) Percentage of myeloid cell ($CD45^+$ $CD11b^+$) retrieved from SO with different monocyte numbers embedded at day 0. Data are presented as the mean ± SD (where n = 2 replicates) of the number of myeloid cells across time points (Days 7, 14, 21; total n = 20 SOs from 2 independent experiments).

(C) Percentage of myeloid cell ($CD45^+$ $CD11b^+$) retrieved from synovial organoids (SO) seeded with 100K monocytes, showing the number of macrophages at different time points. Bar plots show the mean ± SEM % of myeloid cells within total SO cell number. Data from n = 3–4 synovial organoids across 3 independent experiments.

(D) Bar plots showing the mean ± SEM of cell viability from n = 6 synovial organoids in 2 independent experiments.

(E) Representative confocal microscopy images (40x) showing IF staining for CD31 (red), macrophages (CD45, green) and nuclei stained with DAPI (blue) from organoids with FLS derived from biopsies of n=6 patients with active RA at different time points. The insert shows enlarged region of SO. Scale bars, 100 µm.

Supplementary Figure 4 related to Figure 4

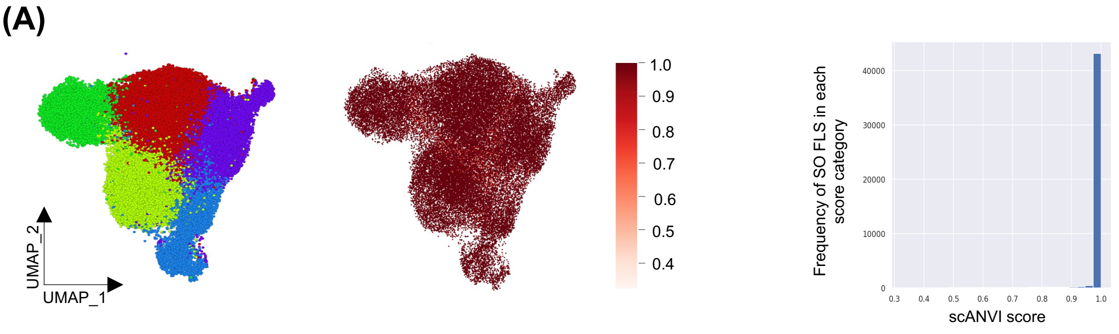

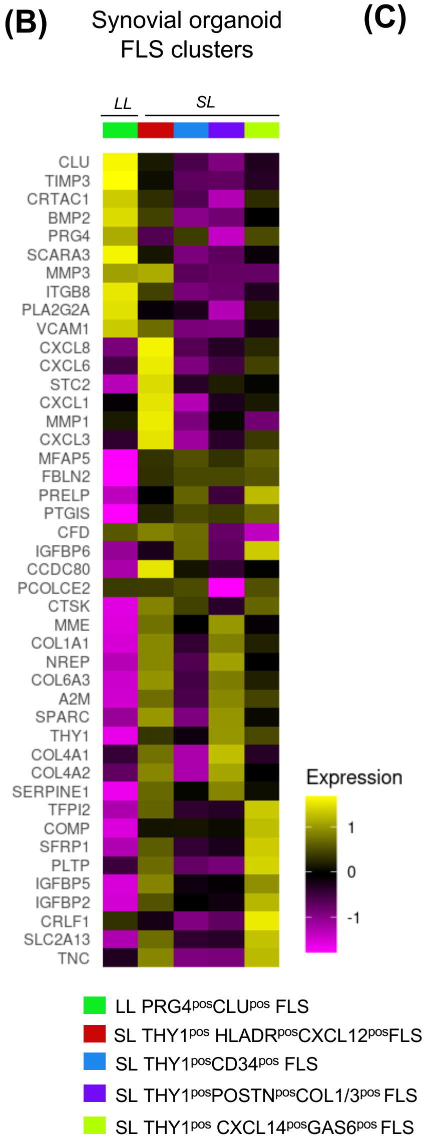

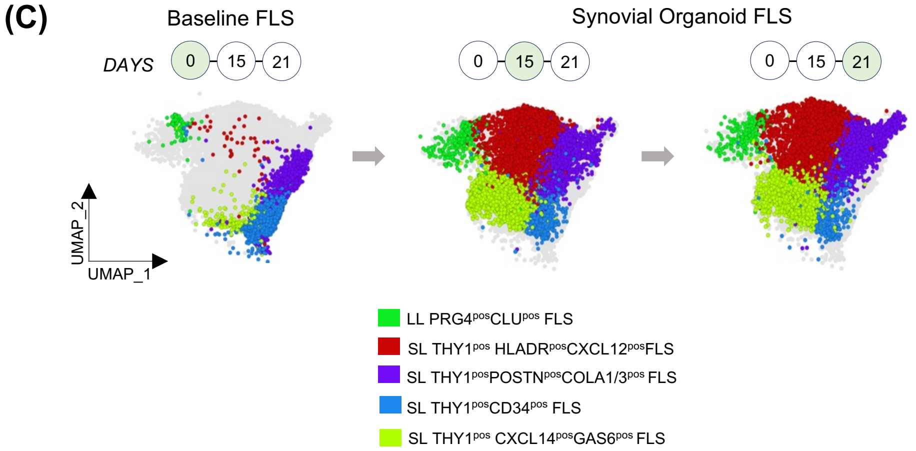

Supplementary Figure 4 Legend. Synovial organoid FLS cluster composition resembles synovial tissue.

(A) UMAP visualization of integrated single-cell data of synovial organoid (SO) and synovial tissue FLS and respective scANVI prediction score (score_scanvi), as feature plot or frequency plot. The score reflects the model's confidence (0–1) in cell-type label assignments, with higher values indicating greater confidence in the predicted cell identity of SO cells.

(B) Heatmap visualizing the gene expression of the top 10 marker genes of SO FLS clusters. Differentially expressed (DE) genes were determined using MAST and were considered significant if expressed in more than 30% of cells in the appropriate cluster with adjusted $p < 0.05$ after Bonferroni correction for multiple comparison, average logFC≥0.5.

(C) UMAP visualization of baseline fibroblasts and SO fibroblast clusters at day 15 and at day 21 (n = 4 technical replicates) as well as a reference tissue FLS. SO=synovial organoids

Supplementary Figure 5 related to Figure 4

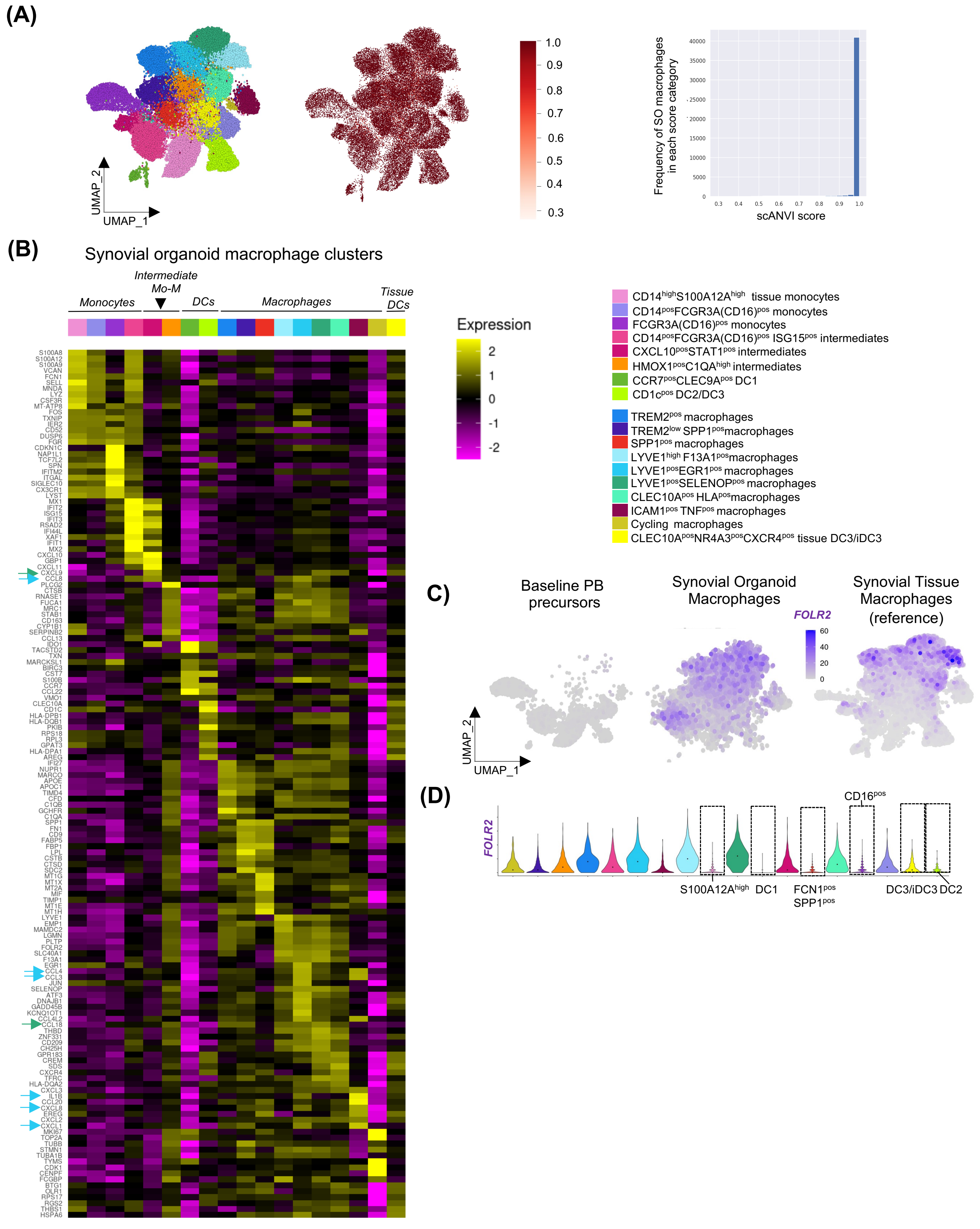

Supplementary Figure 5 Legend. Synovial organoid macrophages (SOM) resemble synovial tissue macrophage (STM).

(A) UMAP visualization of integrated single-cell data of synovial organoid (SO) and synovial tissue myeloid cells and respective scANVI prediction score (score_scanvi), as feature plot or frequency plot. The score reflects the model's confidence (0–1) in cell-type label assignments, with higher values indicating greater confidence in the predicted cell identity of SO cells.

(B) Heatmap visualizing the gene expression of the top 10 marker genes of SO myeloid cells clusters. Differentially expressed (DE) genes were determined using MAST and were considered significant if expressed in more than 30% of cells in the appropriate cluster with adjusted $p < 0.05$ after Bonferroni correction for multiple comparison, average logFC≥0.5.

(C) UMAP feature plot showing the expression levels of the tissue macrophage marker FOLR2 in blood precursors, synovial organoids and in reference synovial tissue macrophages.

(D) Violin plots showing FOLR2 expression in synovial organoid myeloid cell clusters. Dotted frames indicate clusters negative for FOLR2 expression. Arrows indicate gene of interest in perivascular LYVE1^{pos} clusters. SO=synovial organoids.

Supplementary Figure 6 related to Figure 4

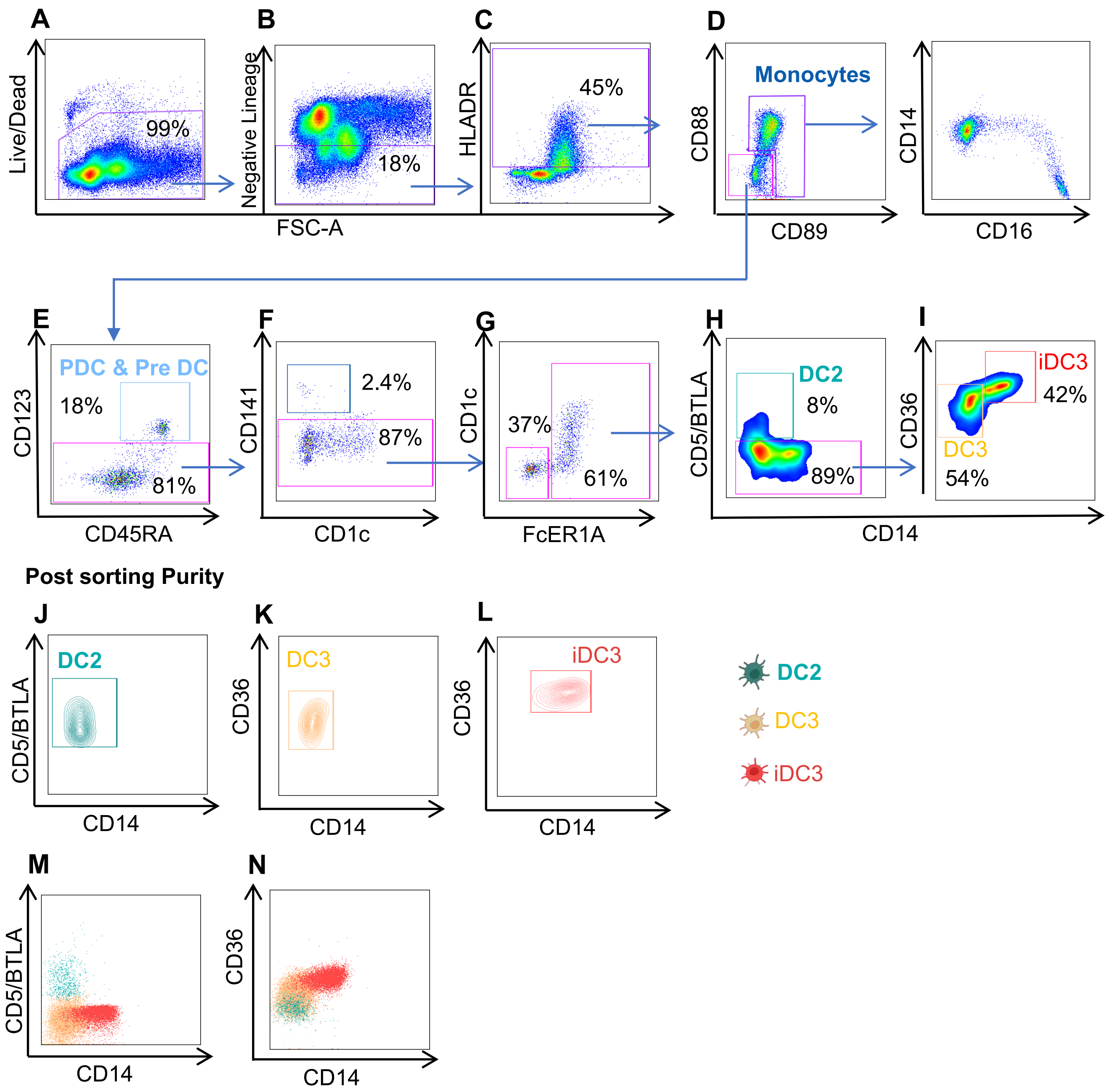

Supplementary Figure 6 Legend. Representative gating strategy for the sorting of healthy PB myeloid populations (monocytes, DC2, DC3 and iDC3) prior to SNP-based organoid generation.

Live cells were gated (A), lineage-positive CD3, CD19, CD15, CD117, CD56 cells were excluded (B), and HLA-DR-positive cells were gated (C). Monocyte were gated based on CD88 and CD89 expression (D). After exclusion of PDC, Pre-DC, and DC1 (E & F), myeloid DCs were gated based on CD1c and FcER1A expression (G). DC2 cells were sorted based on CD5/BTLA (H), while DC3 and iDC3 based on distinct expression of CD14 and CD36 (I). J-N validate the post-sorting purity. All the gates were based on unstained cells or Fluorescence Minus One (FMO) control.

Supplementary Figure 7 related to Figure 4

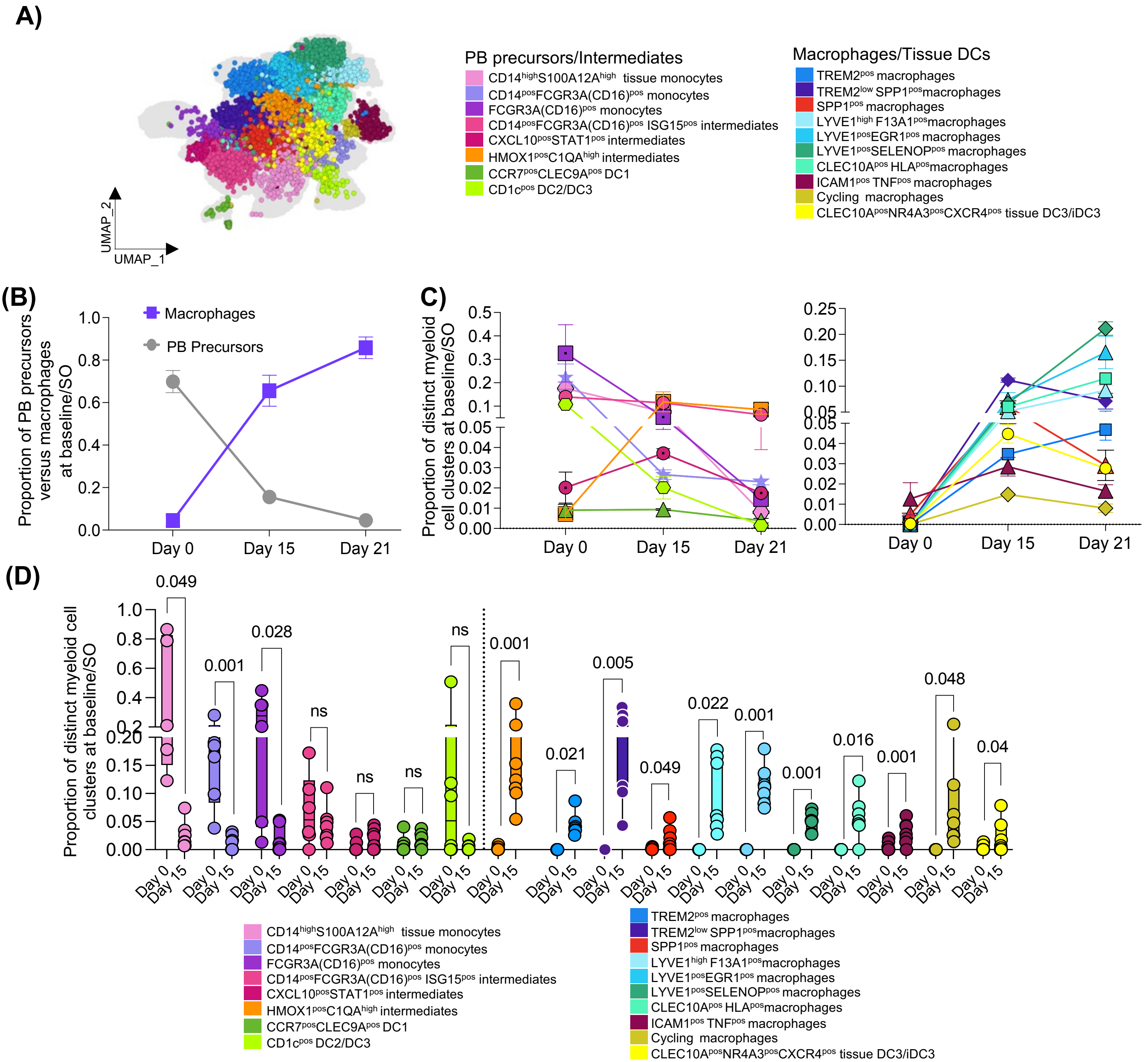

Supplementary Figure 7. Frequency of myeloid cell precursors and distinct myeloid tissue cell clusters derived from them in human synovial organoids (SO).

(A) Overview of integrated SO myeloid clusters as in Fig.4D.

(B) Proportional distribution of PB precursors versus macrophage clusters in SO from data as in (Fig.4J) over time. Cells that mapped with blood cells were defined as PB monocytes/DC and cells that uniquely mapped to synovial tissue were categorized as tissue myeloid cells. Data are presented as a connected Mean ± SD of 2 (baseline) and 4 (SO) technical replicates.

(C) Kinetics of the development of different synovial tissue macrophage clusters from blood monocytes (Day 0) in synovial organoids. Integrated (scANVI) scRNAseq of the myeloid compartment of synovial organoids at days 15 and 21 and matched baseline monocytes (Day 0). Data are presented as mean ± SEM of n = 4 technical replicates.

(D) Proportional distribution of monocyte, dendritic cells and macrophage clusters at baseline (Day 0) and in synovial organoids (SO) at day 15, based on n = 7 experiments as in Figure 4 D-E. Data are presented as the median with the interquartile range. Each dot represents a patient-derived SO. A paired T-test between baseline monocytes (Day 0) and matched myeloid cells at Day 15 organoids, with exact p-values displayed on the graph.

Supplementary Figure 8 related to Figure 6

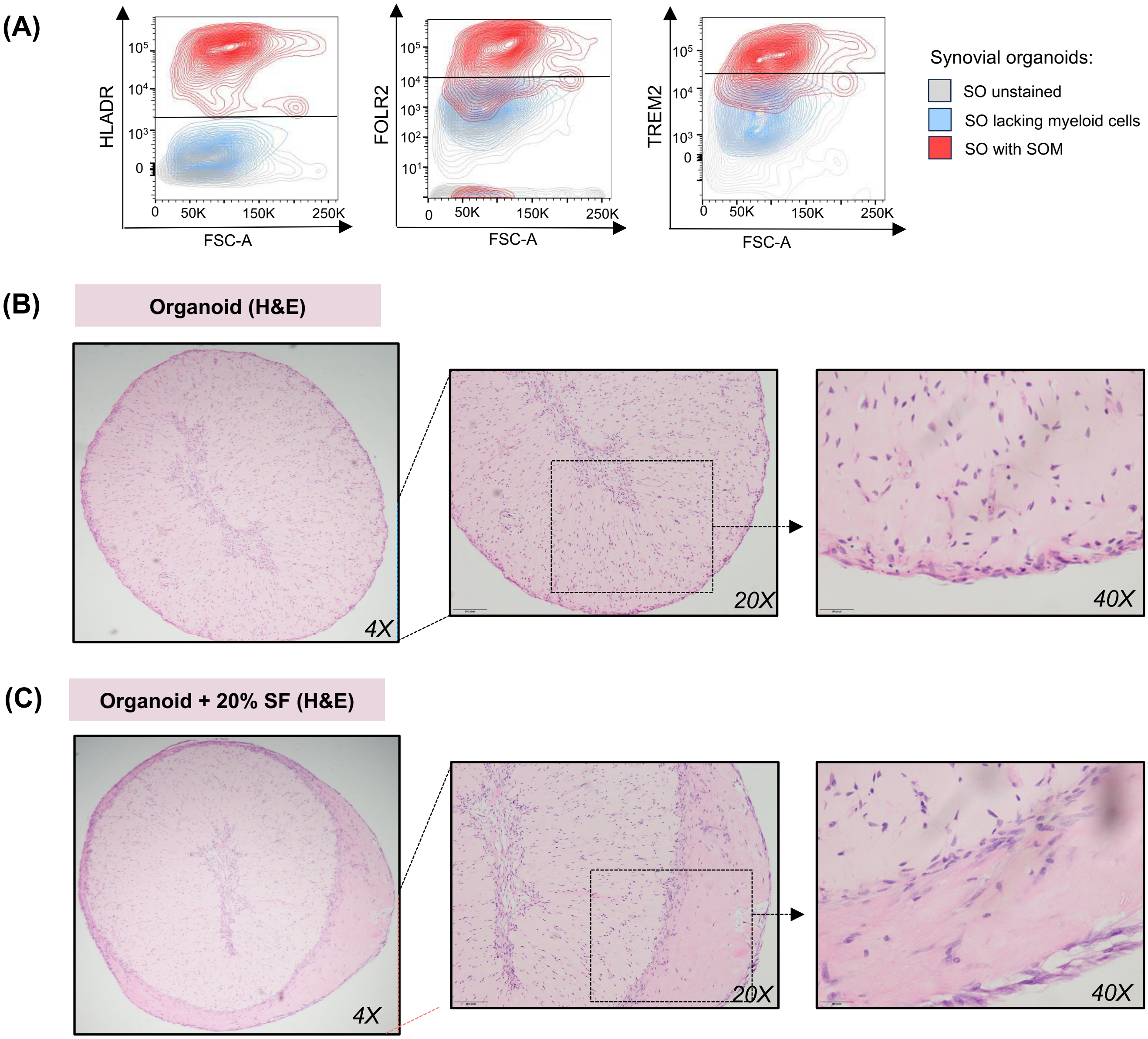

Supplementary Figure 8 Legend. Synovial organoid topography and gating strategy for synovial organoid macrophages.

(A) Representative flow cytometry contour plots showing expression of HLADR, FOLR2 and TREM2 by synovial organoid macrophages. SO were digested at day 15. Unstained SO, grey, SO lacking myeloid cells, blue and SO stained with selected STM markers in red. Data are generated from FLS derived from n=5 RA patients' biopsies with active diseases in five independent experiment with 2-4 technical replicate.

(B-C) The topography of synovial organoids stimulated with synovial fluid (SF). Representative H&E staining of synovial organoid (SO) structure in the absence (B) or in the presence of 20% synovial fluid (SF) for last 7 days of 15 days organoid cultures. Data were generated in 3 independent experiments. SF=pooled cell-free synovial fluid from n=5 patients.
